# Dr. Frederick W. Miller

**Published:** 1908-07

**Authors:** 


DR. FREDERICK W. MILLER, of Dover, N. J., died at All Souls' Hospital, Morristown, N. J., June 6, 1908. He graduated at the University of Buffalo, Medical Department in 1875.

